# Advances in Natural Antioxidants for Food Improvement

**DOI:** 10.3390/antiox11091825

**Published:** 2022-09-16

**Authors:** María López-Pedrouso, José M. Lorenzo, Daniel Franco

**Affiliations:** 1 Department of Zoology, Genetics and Physical Anthropology, Universidade de Santiago de Compostela, 15872 Santiago de Compostela, Spain; 2 Centro Tecnológico de la Carne de Galicia, Rúa Galicia Nº 4, Parque Tecnológico de Galicia, 32900 San Cibrao das Viñas, Spain; 3 Department of Chemical Engineering, Universidade de Santiago de Compostela, 15782 Santiago de Compostela, Spain

## 1. Natural and Synthetic Antioxidants

In the food industry, antioxidants are natural and synthetic compounds added to neutralize free radicals that deteriorate fats, proteins and cellular DNA, causing rancidity of fats and accelerating the ageing process, which lead to undesirable smells and tastes. The most commonly used synthetic phenolic antioxidants are butylated hydroxyanisole (BHA), butylated hydroxytoluene (BHT), tertiary butylhydroquinone (TBHQ) and propyl gallate (PG). However, large adverse health effects are associated with their use, including carcinogenicity, cytotoxicity, oxidative stress induction and endocrine disruption [1,2,3]. Therefore, these additives are very controversial and there is a broad consensus on their partial or total replacement. A strong growth trend in the search for natural antioxidants has already been established.

The antioxidant activity of plant extracts is mainly evaluated by in vitro chemical assays. Overall, the large amount of in vitro antioxidant assays can be divided into the following two categories: electron transfer (SET) assays based on redox reactions and hydrogen atom transfer (HAT) assays based on radical scavenging ability. The antioxidant capacity of a molecule is measured by its ability as a reducing compound by donating a single electron or hydrogen atom, which is strongly affected by the solvent of extraction, pH and other conditions. Among these, ORAC assay as HAT assay is the preferred choice in the food field for multiple ingredients and complex reaction kinetics, even though its antioxidant activity is only measured against peroxyl radicals [4,5]. Additionally, ABTS and DPPH could be used to assess antioxidant activity as SET assays. The main challenges in this field are focused on searching for more reliable methods, in addition to knowledge on their bioavailability and antioxidant capacity in the human body.

## 2. Novel Sources of Natural Antioxidants

For many years, traditional extracts from plants, spices and culinary herbs have been added to enhance flavor and increase the food shelf life. These extracts are rich in high-antioxidant activity compounds, such as polyphenols (phenolic acids, flavonoids, anthocyanins, lignans and stilbenes), carotenoids (xanthophylls and carotenes) and vitamins (vitamin E and C). These antioxidants have important applications in functional food development due to their beneficial effects on human health. The search for novel and natural antioxidants for new products is mandatory considering our modern lifestyle. Ensuring high sensorial quality and food preservation are the major challenges in the present times. In this Special Issue, several authors have investigated different compounds from vegetal sources as novel antioxidants. For instance, the use of terpenoids and polyphenols in the prevention of lipid oxidation of meat, fish or vegetable foodstuff with different amounts of saturated/polyunsaturated fat is widely studied. These compounds were revised using numerous manuscripts to draw general conclusions by Gutiérrez-Del-Río et al. [6]. A study that employed powdered leaves of matcha green tea and moringa to white chocolate during the tempering process improved its antioxidant capacity. The authors attributed this increase to the high amounts of polyphenols in green tea, as shown in [7]. In addition, mango fruits, as a source of bioactive compounds, and the pulp, peel and kernel of different cultivars have been studied. The findings showed that pulp had a great concentration of phytosterols, but also peels had a large quantity of tocopherols, carotenoids, and chlorophylls and kernels had phenolic compounds [8]. Furthermore, it is known that during fruit ripening, the antioxidant concentration in the pulp, peel and seed suffer changes and is difficult to optimize. This effect was studied in phlegrean Mandarin (*Citrus reticulata* Blanco) and the researchers reported that ripe fruits gave rise to the highest activity in peel and seeds [9]. In this line, in a vegetal organism, the balance of reactive oxygen species (ROS) and antioxidant capacity of the cell can be modified. Indeed, fruit ripening is frequently associated with the formation and emission of ROS and melatonin, delaying postharvest ripening of several horticultural crops, such as papaya. A study proved that melatonin enhanced the antioxidant activity and decreased fruit oxidative injury in postharvest papaya fruit [10].

Moreover, different mint species were investigated to compare the drug yield, antioxidant properties and essential oil quality under different extraction conditions. The most noteworthy result was that methanolic and ethanolic extracts from pineapple and grapefruit mint showed the highest antioxidative capacity [11]. In line with essential oils, novel vegetal sources have been proposed to increase food shelf-life, such as oil lemongrass essential oil, which contains relevant terpenes, responsible for antioxidant activity. However, their stability and volatility at high temperatures would suggest employing innovative technologies, including microencapsulation, nanoemulsion and edible films, to add them to foods [12]. On the other hand, other less studied sources of antioxidant compounds have been presented in this Special Issue. For instance, kombucha tea is a not well-known fermented beverage and consists of plant liquids (tea, juices and herb extracts) and a symbiotic culture of bacteria and yeasts. However, it has been demonstrated that it has a high content of bioactive compounds, and strong antioxidant and antimicrobial properties [13]. Furthermore, antioxidant peptides from animal byproducts are currently being investigated. Indeed, porcine liver hydrolysates with alcalase and bromelain enzymes showed antioxidant activity and seventeen peptides were pointed out as potential candidates due to their high correlation with antioxidant activity [14].

## 3. Extraction Methods of Natural Antioxidants

The extraction of natural antioxidants is currently being developed using green non-conventional methods to reduce the usage of organic solvents in an energy-efficient and economically sustainable way. The most relevant methods include extraction assisted by ultrasounds, microwave, pressured liquid, enzyme hydrolysis, supercritical fluids, high hydrostatic pressure, pulsed electric field and high voltage electrical discharges [15]. Several articles in this Special Issue are focused on the optimization of the extraction process.

Polyphenol extraction from rye flour that employed six different solvents (water, methanol, ethanol, acidified methanol, benzene and acetone) was assayed. The results indicated that the maximum antioxidant capacity was shown in water extracts, which is positive from an environmental and human consumption point of view [16]. Another friendly environmental process, known as the enzymatic process, was applied for vegetable juice inoculated with two lactic acid bacteria from kimchi. The results indicated that six metabolites that correlated with antioxidant activity were increased [17].

Another strategy to diminish the extraction costs consists of the use of waste and residues from the agro-industrial field [18]. Relevant technological advances in this field were studied and discussed in this Special Issue. For example, important agri-food wastes, such as potato peels, could be used to obtain bioactive compounds with added value. The recovery of polyphenols (phenolic acids and flavonoids) from skins would diminish environmental and economic problems, as reviewed by Rodríguez-Martínez et al. (2021) [19]. Avocado peels were also analyzed and bioactive molecules were extracted by ultrasounds with mixtures of ethanol and water. Phenolic acids (hydroxybenzoic and hydroxycinnamic acids) and flavonoids (flavanols, flavanonols, flavones, flavanones and chalcone), phenylethanoids and lignans were detected in peels [20]. In chocolate manufacturing, large amounts of by-products are produced from cocoa, which contains bioactive compounds, such as methylxanthines, flavonoids, procyanidins. Specifically, shell and pod husk flours were investigated and were found to contain several bioactive polyphenolic compounds [21,22]. The table olive industry produces a great amount of wastewater that is usually treated under alkaline conditions, followed by washing stages for stabilization. It has been demonstrated that these streams could be used to recover phenolic substances [23]. Finally, the beer industry also produces a large amount of by-products, from the brewing process. Cereals (maize, rice, wheat, oats, rye or sorghum) mixed with barley malt are subjected to xylanase treatment and fermentation of *lactiplantibacillus plantarum,* releasing polyphenols. Therefore, this material could be used to obtain enriched pasta with high antioxidant activity [24].

## 4. Technological Advances in the Incorporation of Natural Antioxidants in Processed Foods

The addition of natural antioxidant compounds into foodstuff to extend the shelf-life and to improve the sensorial quality is currently being developed for the food industry. Several technological advances in this topic were reported in this Special Issue. In several cases, the natural antioxidant is included in the food formulation or in the packaging. More examples of the first option are presented. For instance, red seaweed has shown antioxidant and antimicrobial properties, as well as relevant textural properties associated with phycocolloids (agar and carrageenan). The protective effect of flour from *Gelidium* sp. on the nutritional and sensorial values of thermally treated seafood was monitored and assessed [25]. Other hydrocolloid-based foods, such as surimi protein gel, together with their additives, were also studied in terms of retarding protein oxidation and denaturation. Gelling and structural properties of surimi gels were reviewed in detail and high-pressure processing, ultrasonication, microwave, and ultraviolet treatments were considered [26]. In the case of chilled seafood, the introduction of preservative compounds in flake ice systems was proposed. General aspects of adding plant-extract compounds, low-molecular-weight organic acids or macroalgae extracts were collected and discussed in a very relevant review by Aubourg [27]. For the second option related to plastic containers, citrus by-products could be successfully introduced in food packaging through plastic impregnation and this could be used to preserve fresh food [28].

Anthocyanins are colored and heat-sensitive compounds with high antioxidant activity. The stabilization by linkage to other molecules via glycosyl acylation with organic acids, as well as molecular mechanisms and kinetics of their degradation, was reviewed by Oancea [29]. In line with this, the stability and solubility of the natural antioxidants were analyzed to enhance their absorption and interaction in the human body. For example, the flavonoid naringin from citrus fruits was esterified with oleic acid, improving its solubility and stability [30]. 

Finally, cutting-edge technology in this field could be used to maintain different fresh-cut products. For example, the ascorbate Bluetooth analyzer, as a screen-printed sensor-based device for ascorbic acid detection, was developed to monitor problems during the processing or transport of products and to detect losses in the cold chain [31].

Of great importance is the use of antioxidants in the meat industry to prevent protein oxidation and lipid peroxidation. Additionally, active packaging as promising technology is being developed to extend the shelf life of meat products [32]. Moreover, there is a great concern about our health and chemical compounds are rejected by consumers. To develop healthy products, antioxidant components are also of great importance for their health benefits, but more work is needed. Leaves from several plant species have a great concentration of polyphenols and their usage has been considered in the elaboration of meat products. Meat formulations of frankfurters, ground meat, hamburgers, meatballs and sausages could include leaves of black and green tea, black currant, blueberry, cilantro, etc., as reviewed by Velázquez et al. [33]. Berry extracts were added to pork patties, resulting in lower lipid oxidation rates measured as TBARS values and a higher shelf life. Additionally, these extracts did not decrease the sensorial quality in terms of appearance, raw odor, cooked odor, texture, taste and overall impression [34]. Extracts of moringa oleifera and olive olea europaea L. were assayed in chicken burgers, slowing the deterioration of the meat in terms of peroxide value and lipid oxidation. Olive leaves reduced protein hydrolysis and total volatile nitrogen content but moringa leaves had less impact on sensory attributes of chicken burgers [35].

In cured meat products, nitrite and nitrate treatments must be minimized due to potential nitrosamine formation, and for that reason, acid whey could be employed in the fermentation of pork bacon. However, nitrites and nitrates showed higher protection of lipid oxidation, according to the fatty acid profiles and TBARS index [36].
